# Transcriptomic and Metabolomic Insights into Growth Heterosis of *Crassostrea hongkongensis* Hybrids

**DOI:** 10.3390/ijms27156631

**Published:** 2026-07-25

**Authors:** Tuo Yao, Jie Lu, Shengli Fu, Mei Xie, Xiaodi Wang, Qisheng Wu, Yue Ning, Xiang Guo, Jun Ye, Hui Ge, Lingtong Ye

**Affiliations:** 1 Key Laboratory of Efficient Utilization and Processing of Marine Fishery Resources of Hainan Province, Sanya Tropical Fisheries Research Institute, Sanya 572018, China; yaotuo@scsfri.ac.cn (T.Y.); lujie@scsfri.ac.cn (J.L.); fushengli@scsfri.ac.cn (S.F.); xiemei0918@163.com (M.X.); 15650023938@163.com (X.W.); 2 Key Laboratory of Cultivation and High-Value Utilization of Marine Organisms in Fujian Province, Fujian Collaborative Innovation Center for Exploitation and Utilization of Marine Biological Resources, Fisheries Research Institute of Fujian, Xiamen 361013, China; wqsljwjx@163.com (Q.W.); ningyfjfri@163.com (Y.N.); guoxiang432@163.com (X.G.); yj3316@163.com (J.Y.); 3 College of Fisheries and Life Science, Dalian Ocean University, Dalian 116023, China; 4 Key Laboratory of South China Sea Fishery Resources Exploitation & Utilization, Ministry of Agriculture and Rural Affairs, South China Sea Fisheries Research Institute, Chinese Academy of Fishery Sciences, Guangzhou 510300, China; 5 Tropical Aquaculture Research and Development Center, South China Sea Fisheries Research Institute, Chinese Academy of Fishery Sciences, Sanya 572018, China

**Keywords:** growth difference, transcriptome, metabolome, *Crassostrea hongkongensis*

## Abstract

*Crassostrea hongkongensis* is an economically important mariculture species in southern China, and hybrid breeding has been applied to improve its growth traits. However, the molecular mechanisms underlying growth heterosis remain poorly understood. In this study, inter-population hybrid (HG) and intra-population (IG) groups of *C. hongkongensis* were compared using integrated transcriptomic and metabolomic analyses to investigate the regulatory basis of growth heterosis. Shell length and shell height were significantly greater in HG than in IG (*p* < 0.01). Transcriptomic analysis identified 474 false discovery rate (FDR)-supported significant differentially expressed genes (DEGs) (233 upregulated and 241 downregulated), which were treated as the primary statistically robust findings; a broader set of 3161 candidate DEGs, including genes associated with growth regulation and shell biomineralization, was retained for exploratory functional and multi-omics analyses. Metabolomic analysis detected 345 differential metabolites (DMs), which were mainly enriched in energy metabolism, nucleotide metabolism, and arachidonic acid metabolism. Exploratory pathway-level multi-omics integration revealed significant concordance between the transcriptomic and metabolomic profiles (M^2^ = 0.1962, *p* = 0.001) and highlighted the tricarboxylic acid cycle, oxidative phosphorylation, branched-chain amino acid degradation, nucleotide metabolism, and arachidonic acid metabolism. These findings suggest that growth heterosis in *C. hongkongensis* is associated with altered energy metabolism, biosynthetic processes, shell formation-related pathways, and potential changes in the growth–defense balance, providing molecular insights for genetic improvement and selective breeding.

## 1. Introduction

*Crassostrea hongkongensis* is an economically important shellfish species widely cultured in the southern coastal areas of China. With its rapid growth, strong environmental adaptability, high nutritional value and large market demand, it has become one of the pillar industries of mariculture in South China [1,2]. In recent years, the *C. hongkongensis* industry has been confronted with multiple challenges, including slow growth, inbreeding depression, reduced stress resistance and frequent mass mortality in summer [3]. These problems have severely restricted the sustainable development of the whole industry. Meanwhile, southern coastal regions are characterized by frequent typhoons and increasing extreme weather events, and the relatively long growth cycle further elevates aquaculture risks [4,5]. Growth performance is among the most critical economic traits in aquaculture, as it is directly linked to culture duration, survival rate, production yield and economic benefits [6,7]. Therefore, developing new fast-growing strains of *C. hongkongensis* has become a key strategy to break through industrial bottlenecks and promote the high-quality development of oyster farming.

In the genetic breeding of aquatic animals, hybridization is one of the most classic and effective approaches to exploit heterosis and rapidly improve economically important traits. Heterosis refers to the phenomenon whereby hybrid progenies exhibit superior performance relative to their parents in growth, survival, stress resistance and other valuable traits [8,9], and has been widely applied in the genetic improvement of aquatic animals [10,11,12,13]. Numerous studies have confirmed that hybridization can effectively alleviate inbreeding depression and significantly enhance growth rate, yield and environmental adaptability in various mollusks, including oysters, scallops, clams, and abalones [14,15,16,17]. In our previous work, we conducted hybridization breeding in *C. hongkongensis* and obtained hybrid progeny with obvious growth heterosis. However, the molecular basis and metabolic regulatory pathways underlying such heterosis remain poorly understood, which seriously limits the efficient application of hybridization and hinders the targeted breeding of elite strains of *C. hongkongensis*.

With the rapid development of high-throughput omics technologies, integrated transcriptomic and metabolomic analysis has become a powerful strategy to dissect the molecular mechanisms of complex economic traits [18,19]. Transcriptomics reveals changes in functional gene expression and related regulatory pathways, and helps identify key regulatory genes and signaling patterns [20]. Metabolomics reflects the final physiological state and phenotypic responses of organisms, directly characterizing energy supply, anabolism and stress adaptation, thus acting as a critical bridge connecting gene expression and phenotypic variation [21]. The combination of transcriptomics and metabolomics enables systematic interpretation of the molecular and metabolic basis of phenotypic differences at multiple levels, overcoming the limitations of single-omics analyses. This integrated approach has been successfully applied to explore growth regulatory mechanisms in a variety of aquatic animals [22,23,24,25,26]. Nevertheless, systematic multi-omics evidence explaining growth heterosis in *C. hongkongensis* remains scarce, and the underlying regulatory mechanisms are largely uncharted.

Hybridization breeding in *C. hongkongensis* has been explored at both intraspecific and interspecific levels. Reciprocal crosses between the Zhuhai and Maowei Sea stocks exhibited enhanced growth and survival [27], while interspecific crosses involving *Crassostrea ariakensis* and *Crassostrea sikamea* produced viable offspring with varying advantages in growth, survival, and environmental tolerance [28,29]. However, these studies primarily evaluated cross-compatibility and phenotypic performance. Molecular studies in other oyster systems have associated heterosis with non-additive gene expression and altered protein metabolism [30], regulation of energy metabolism, osmoregulation, immunity, and apoptosis [31], accelerated aerobic energy metabolism and maternal effects [32], and non-additive and overdominant gene expression associated with thermal-resistance heterosis [33]. Nevertheless, the coordinated transcriptomic and metabolic mechanisms underlying inter-population growth heterosis in *C. hongkongensis* remain poorly understood.

Therefore, the novelty of the present study lies in integrating transcriptomic and metabolomic analyses to compare the inter-population hybrid (HG) and intra-population (IG) groups of *C. hongkongensis*. Specifically, we sought to determine whether growth heterosis in HG is accompanied by coordinated transcriptional and metabolic changes and to identify the biological pathways associated with these changes. We hypothesized that the superior growth of HG would be associated with coordinated transcriptional and metabolic changes in pathways related to energy metabolism, nucleotide metabolism, immune regulation, and shell biomineralization. The corresponding null hypothesis was that HG and IG would show no significant differences in growth-related transcriptomic or metabolomic profiles. This study provides new insights into the molecular basis of growth heterosis and supports the genetic improvement of *C. hongkongensis*.

## 2. Results

### 2.1. Growth Performance of C. hongkongensis

**Figure 1:**
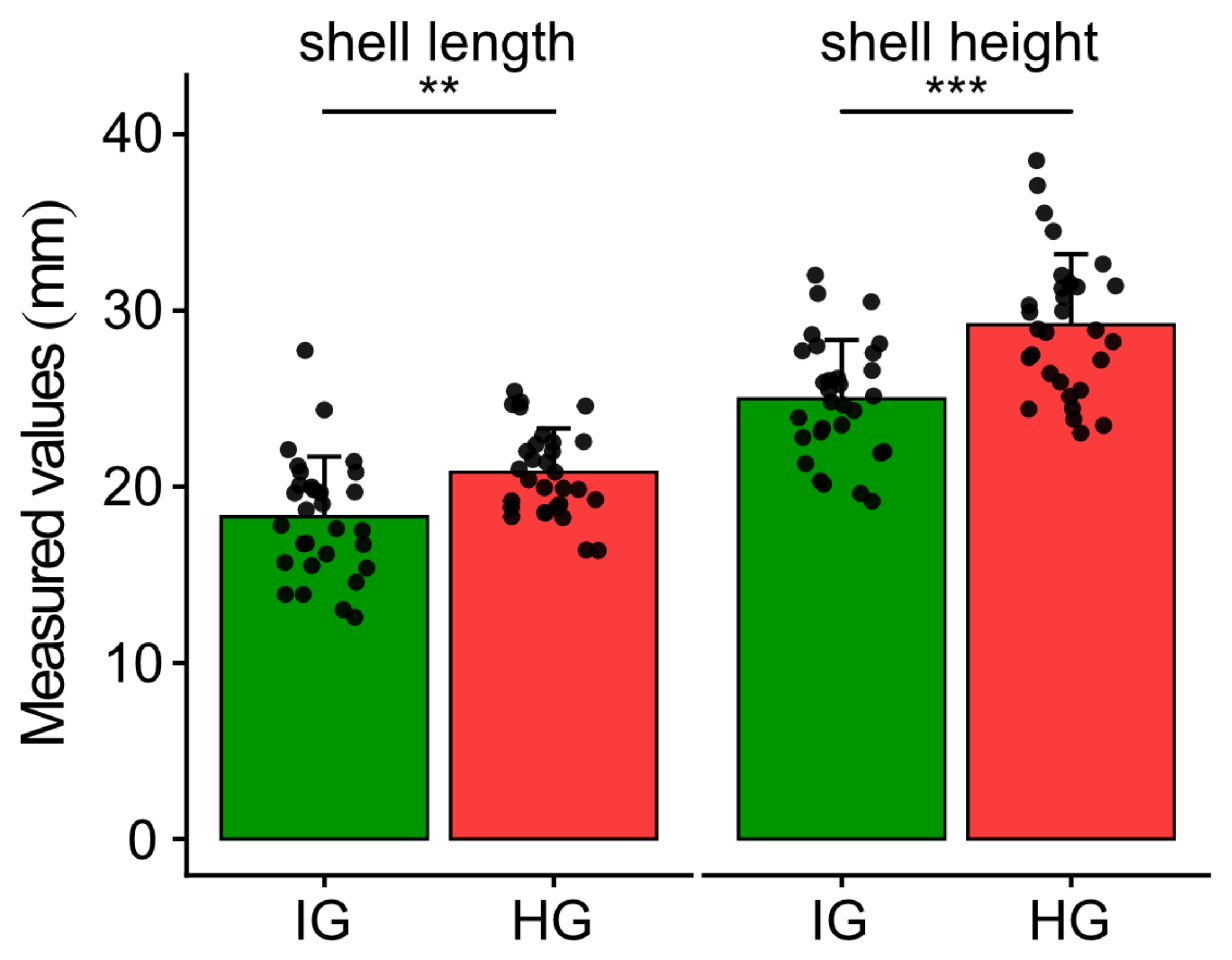
Growth performance of the inter-population hybrid group (HG) and intra-population group (IG) of *Crassostrea hongkongensis*. Student’s *t*-test was used to compare growth traits between the two groups. ** indicates *p* < 0.01, and *** indicates *p* < 0.001.

At six months post-settlement, shell height was compared among the four mating groups generated using a complete diallel crossing design. Both reciprocal hybrid groups derived from the Qinzhou (Q) and Shanwei (S) populations (QS and SQ) exhibited exhibited significantly greater shell height than the two intra-population groups (QQ and SS) (*p* < 0.05), whereas no significant differences were detected between QS and SQ or between QQ and SS (*p* > 0.05) (Figure S1), indicating that the growth advantage was evident in both cross directions. Given the comparable growth performance of the reciprocal hybrid groups and the absence of a significant difference between the intra-population groups, SQ and QQ, which showed a pronounced difference in mean shell height, were selected as the representative hybrid group (HG) and intra-population group (IG), respectively, for subsequent multi-omics analyses to characterize the molecular and metabolic changes associated with growth heterosis. The HG group had a shell length of 20.82 ± 2.48 mm and a shell height of 29.19 ± 4.01 mm, whereas the corresponding values in the IG group were 18.30 ± 3.40 mm and 24.98 ± 3.34 mm, respectively. Both shell length and shell height were significantly greater in HG than in IG (Figure 1).

### 2.2. Transcriptomic Analysis

#### 2.2.1. Transcriptome Sequencing and Quality Control

Transcriptome sequencing was performed on 12 pooled mantle tissue samples, including six biological replicates from the IG group and six biological replicates from the HG group. After quality filtering, high-quality clean reads were obtained for subsequent bioinformatic analysis. As shown in Table S1, all samples exhibited high and consistent sequencing quality: the Q20 and Q30 values ranged from 99.37% to 99.43% and 96.79% to 97.04%, respectively, confirming high base-calling accuracy. The GC content was stable across all samples, ranging from 43.00% to 44.28%. The mapping rate of clean reads to the de novo-assembled transcriptome reference ranged from 85.51% to 87.40%, indicating reliable alignment and supporting the suitability of the sequencing data for subsequent transcriptomic analyses.

#### 2.2.2. Identification of Candidate and FDR-Supported Significant DEGs Between the IG and HG Groups

**Figure 2:**
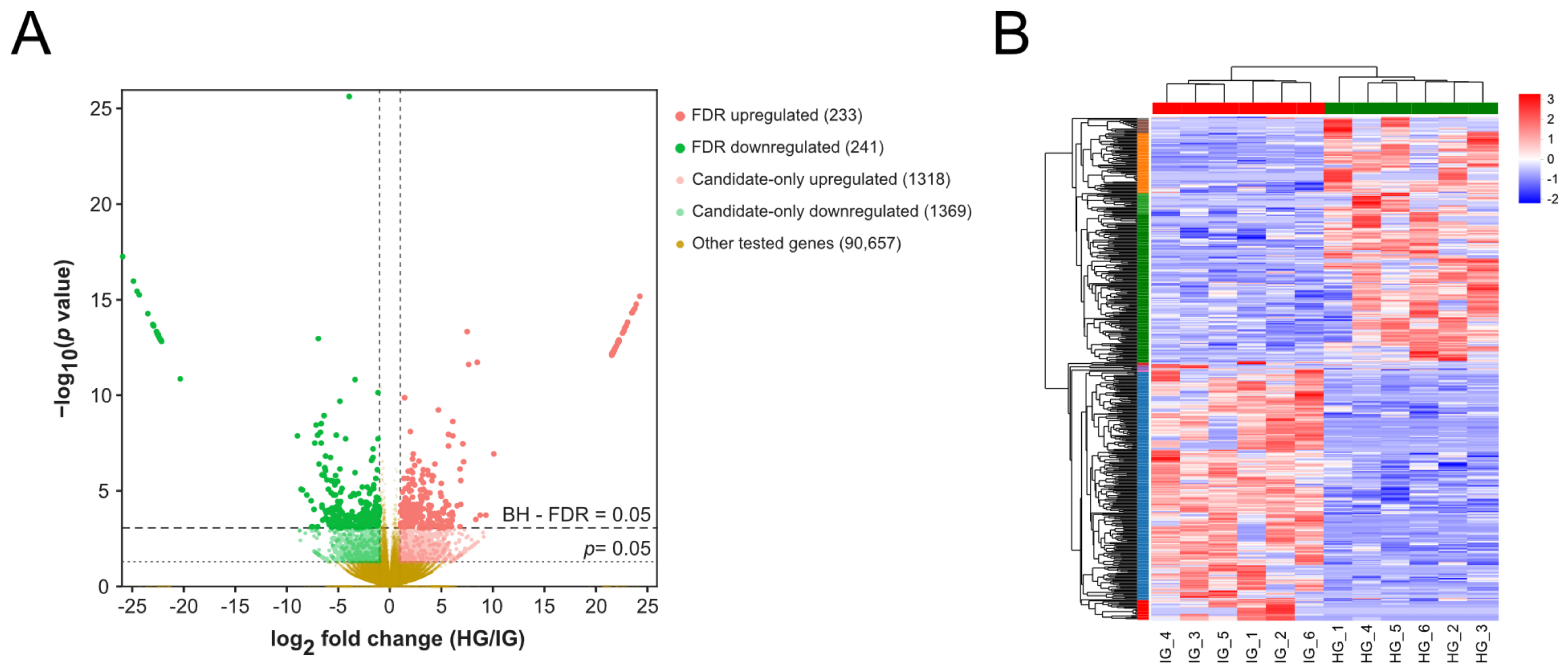
Global gene expression profiles in the mantle tissues of *C. hongkongensis* in the HG and IG groups. (**A**) Volcano plot of all 93,818 tested genes. The y-axis represents −log_10_(*p*), where *p* denotes the unadjusted p value. Dark coral and dark green points indicate 233 upregulated and 241 downregulated FDR-supported significant DEGs, respectively, meeting Benjamini–Hochberg-adjusted *p* < 0.05 and |log_2_FC| ≥ 1. Light coral and light green points indicate 1318 candidate-only upregulated and 1369 candidate-only downregulated genes, respectively, meeting *p* < 0.05 and |log_2_FC| ≥ 1 but not the FDR threshold. Gold points indicate the remaining 90,657 tested genes. The dotted horizontal line indicates *p* = 0.05, and the long-dashed horizontal line indicates the dataset-specific unadjusted-*p*-value rejection boundary (8.6123 × 10^−4^) corresponding to BH-FDR = 0.05. Vertical dashed lines indicate log_2_FC = −1 and +1. (**B**) Hierarchical clustering heatmap of the 3161 candidate DEGs.

Transcriptome analysis revealed distinct transcriptional profiles between the two groups. Among the 93,818 tested genes, Benjamini–Hochberg correction identified 474 false discovery rate (FDR)-supported significant differentially expressed genes (DEGs) at an adjusted *p* value < 0.05 and |log_2_FC| ≥ 1, comprising 233 upregulated and 241 downregulated genes in HG relative to IG (Figure 2A; Table S2). These 474 genes were treated as the primary statistically robust differential-expression findings. Using the exploratory criteria of *p* < 0.05 and |log_2_FC| ≥ 1, a broader set of 3161 candidate DEGs was identified, comprising 1551 upregulated and 1610 downregulated candidates; of these, 2687 were candidate-only DEGs that did not meet the FDR threshold (1318 upregulated and 1369 downregulated). The broader candidate set was retained for exploratory functional, pathway-enrichment, and multi-omics analyses and is provided in Table S2. Hierarchical clustering of the candidate DEG set showed a clear separation of expression profiles between IG and HG (Figure 2B).

**Figure 3:**
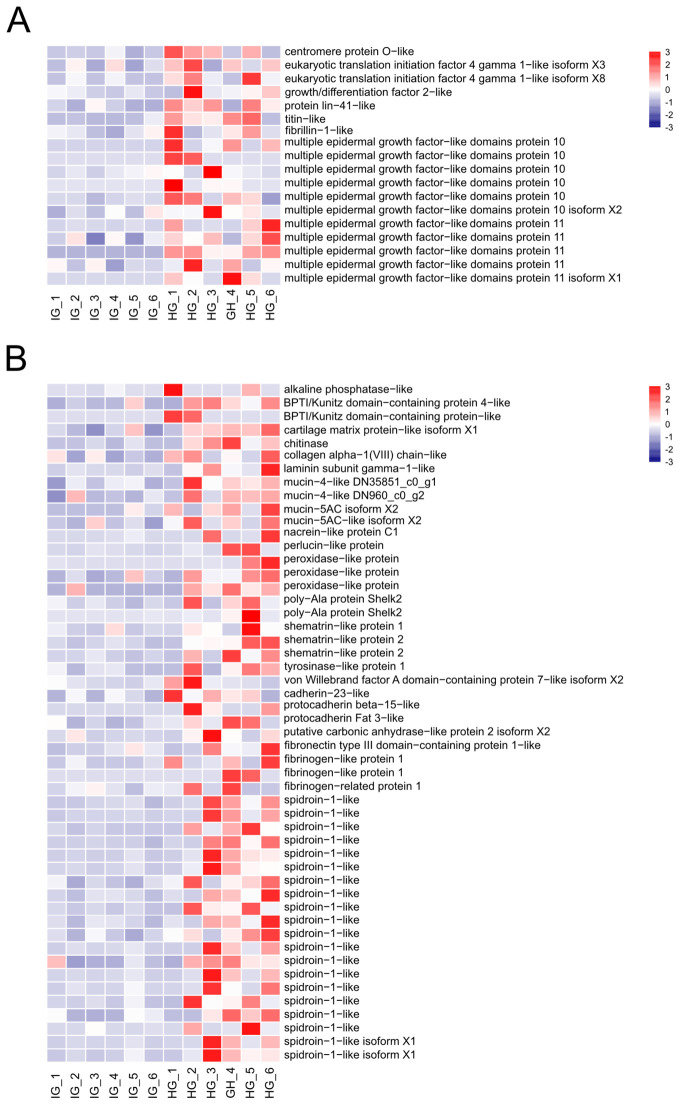
Functional classification and expression profiling heatmaps of candidate DEGs in *C. hongkongensis* between the HG and IG groups. (**A**) Heatmap of candidate DEGs associated with growth regulation. (**B**) Heatmap of candidate DEGs associated with biomineralization.

To explore functional patterns within the broader candidate DEG set in relation to growth heterosis, we conducted targeted expression profiling of two key functional categories closely linked to growth traits: growth regulation and biomineralization. For growth-related genes, growth/differentiation factor 2-like (*GDF2*), multiple epidermal growth factor-like domains protein 10 (*MEGF10*) and protein 11 (*MEGF11*), and other growth-related genes showed higher expression in the HG group under the exploratory candidate-DEG screening criteria (*p* < 0.05 and |log_2_FC| ≥ 1; Figure 3A). For biomineralization-related genes, spidroin-1-like, nacrein-like protein C1, shematrin-like protein 1, and other genes associated with shell formation also predominantly showed higher expression in the HG group (Figure 3B).

#### 2.2.3. RT-qPCR Validation of the DEGs

**Figure 4:**
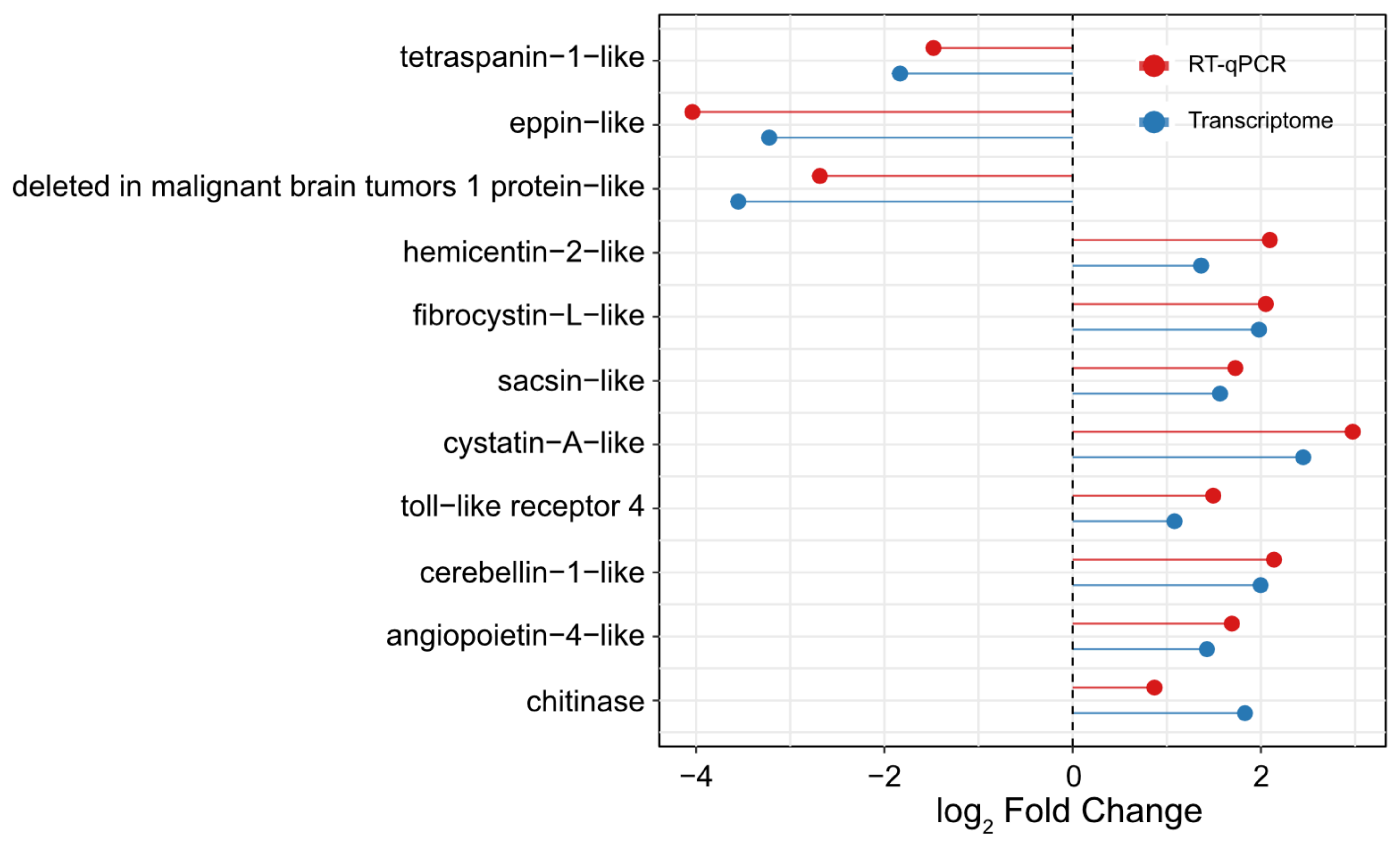
Comparison of gene expression data from RNA-seq and RT-qPCR. The y-axis is the gene name and the x-axis represents the log_2_ fold change in gene expression compared to the IG group.

To assess the reliability of the transcriptome sequencing data, we performed RT-qPCR validation on 11 randomly selected candidate DEGs, including 8 up-regulated genes (chitinase, angiopoietin-4-like, cerebellin-1-like, toll-like receptor 4, cystatin-A-like, sacsin-like, fibrocystin-L-like, hemicentin-2-like) and 3 down-regulated genes (deleted in malignant brain tumors 1 protein-like, eppin-like, tetraspanin-1-like) from the HG group. The RT-qPCR results showed expression patterns consistent with those obtained by RNA-seq (Figure 4). Moreover, Pearson’s correlation analysis based on the log_2_ fold change values revealed a significant positive correlation between the two methods (r = 0.956, *p* < 0.001), supporting the reliability of the RNA-seq results.

### 2.3. Metabolomics Analysis

**Figure 5:**
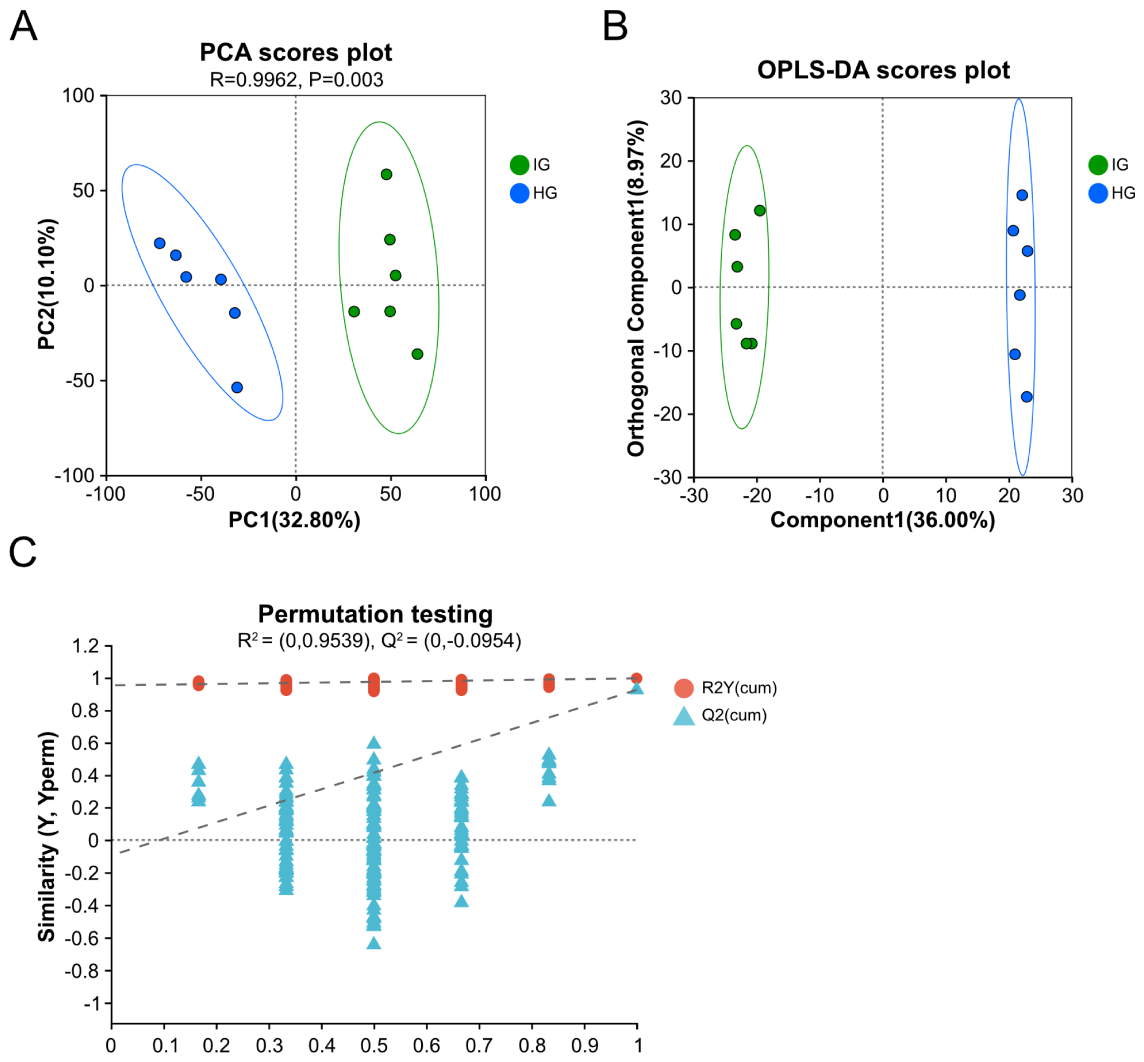
Quality assessment of the combined positive- and negative-ion-mode metabolomic dataset from the HG and IG groups of *C. hongkongensis.* (**A**) PCA score plot showing overall metabolic variation between the two groups. (**B**) OPLS-DA score plot for supervised differentiation of metabolic profiles between HG and IG groups. (**C**) Permutation test of the OPLS-DA model with 200 permutations.

To assess the global metabolic differences between the HG and IG groups, principal component analysis (PCA) and orthogonal partial least squares discriminant analysis (OPLS-DA) were performed on the combined positive- and negative-ion-mode metabolomic dataset. The PCA, OPLS-DA, and subsequent permutation test were all based on this combined dataset. As shown in Figure 5A, the PCA scores plot revealed a clear separation between the HG and IG groups, indicating distinct overall metabolic profiles. Consistent with the PCA results, the OPLS-DA scores plot also showed a clear separation between the two groups (Figure 5B). The OPLS-DA model comprised one predictive component and one orthogonal component and yielded cumulative R^2^X, R^2^Y, and Q^2^ values of 0.450, 0.997, and 0.926, respectively (Table S3). The high Q^2^ value indicated strong predictive ability within the present dataset. Model performance was further assessed using a permutation test with 200 random permutations of the group labels. None of the permuted models yielded R^2^Y or Q^2^ values exceeding those of the original model (0/200; permutation *p* < 0.05). Moreover, the permuted Q^2^ values were substantially lower than the original Q^2^ value, and the Q^2^ regression intercept was negative (−0.0954), further supporting predictive performance beyond random classification (Figure 5C and Table S3). The relatively high R^2^Y intercept (0.9539) was evaluated together with the Q^2^-based permutation results rather than as a standalone measure of model validity. Collectively, the PCA and OPLS-DA results demonstrated distinct metabolic profiles between the HG and IG groups and supported the strong predictive ability of the OPLS-DA model within the present dataset.

**Figure 6:**
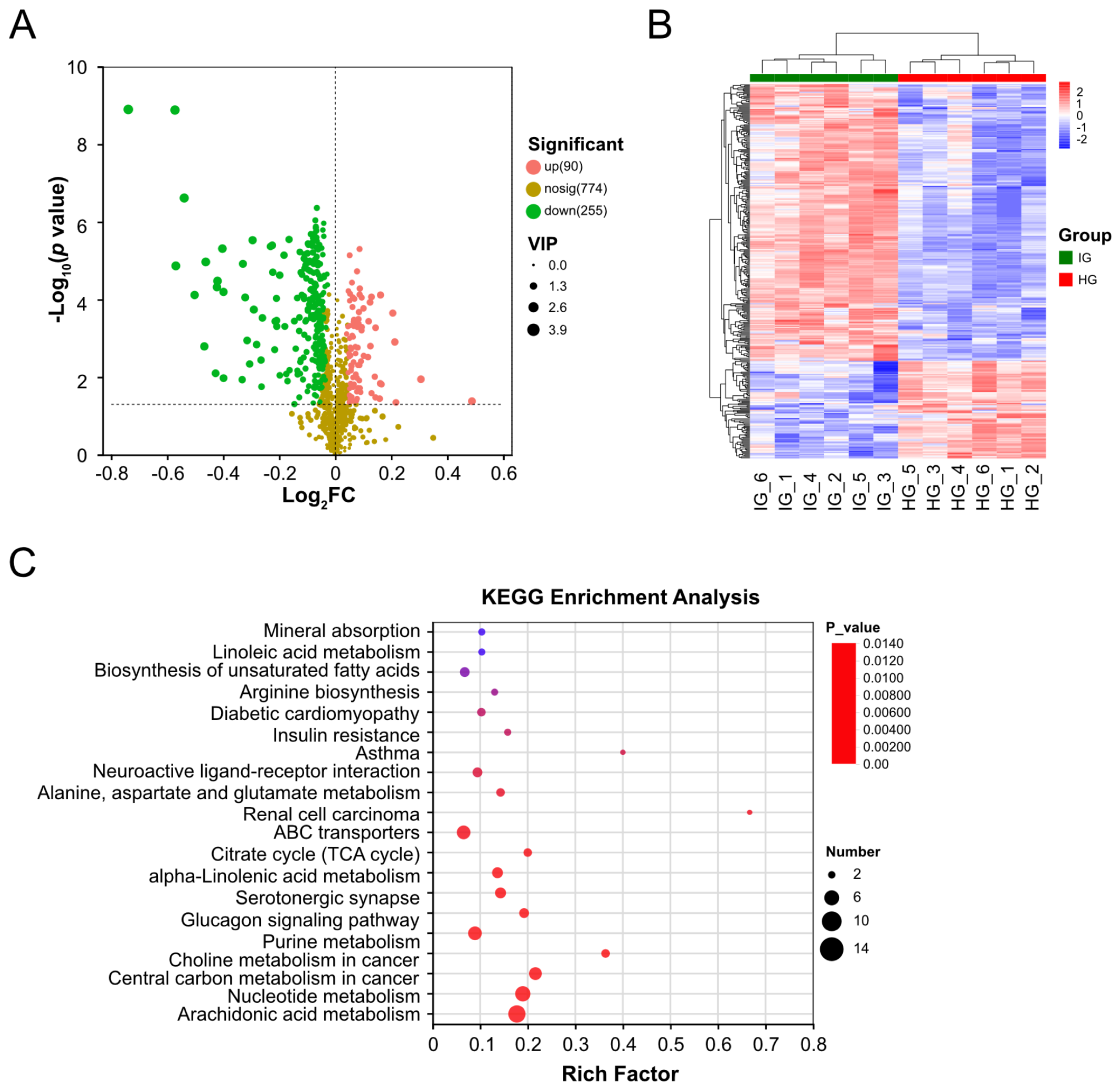
Metabolomic analysis of the HG and IG groups. (**A**) Volcano plot of differential metabolites between the HG and IG groups. (**B**) Hierarchical clustering heatmap of differential metabolites between the HG and IG groups. Red indicates metabolites upregulated in the HG group; blue indicates metabolites upregulated in the IG group. (**C**) KEGG (Kyoto Encyclopedia of Genes and Genomes) pathway enrichment analysis of differential metabolites between the HG and IG groups.

A total of 345 differential metabolites (DMs) were identified between the HG and IG groups (screening thresholds: variable importance in projection (VIP) > 1, *p* < 0.05). Compared with the IG group, 90 DMs showed increased abundance and 255 showed decreased abundance in the HG group (Figure 6A and Table S4). Hierarchical clustering analysis of all DMs was performed to visualize their abundance patterns, and the resulting heatmap showed a clear separation between the HG and IG groups, confirming the distinct metabolic profiles of the two groups (Figure 6B). To elucidate the key metabolic pathways driving growth heterosis in hybrid oysters, we conducted KEGG pathway enrichment analysis on all identified DMs. As shown in Figure 6C, the top 20 enriched pathways were mainly associated with nucleotide metabolism, purine metabolism, the tricarboxylic acid (TCA) cycle, and arachidonic acid metabolism. These findings indicate that these core metabolic pathways likely drive the phenotypic differences between the HG and IG groups of Hong Kong oysters.

### 2.4. Integrative Analysis of Transcriptomic and Metabolomic Data

**Figure 7:**
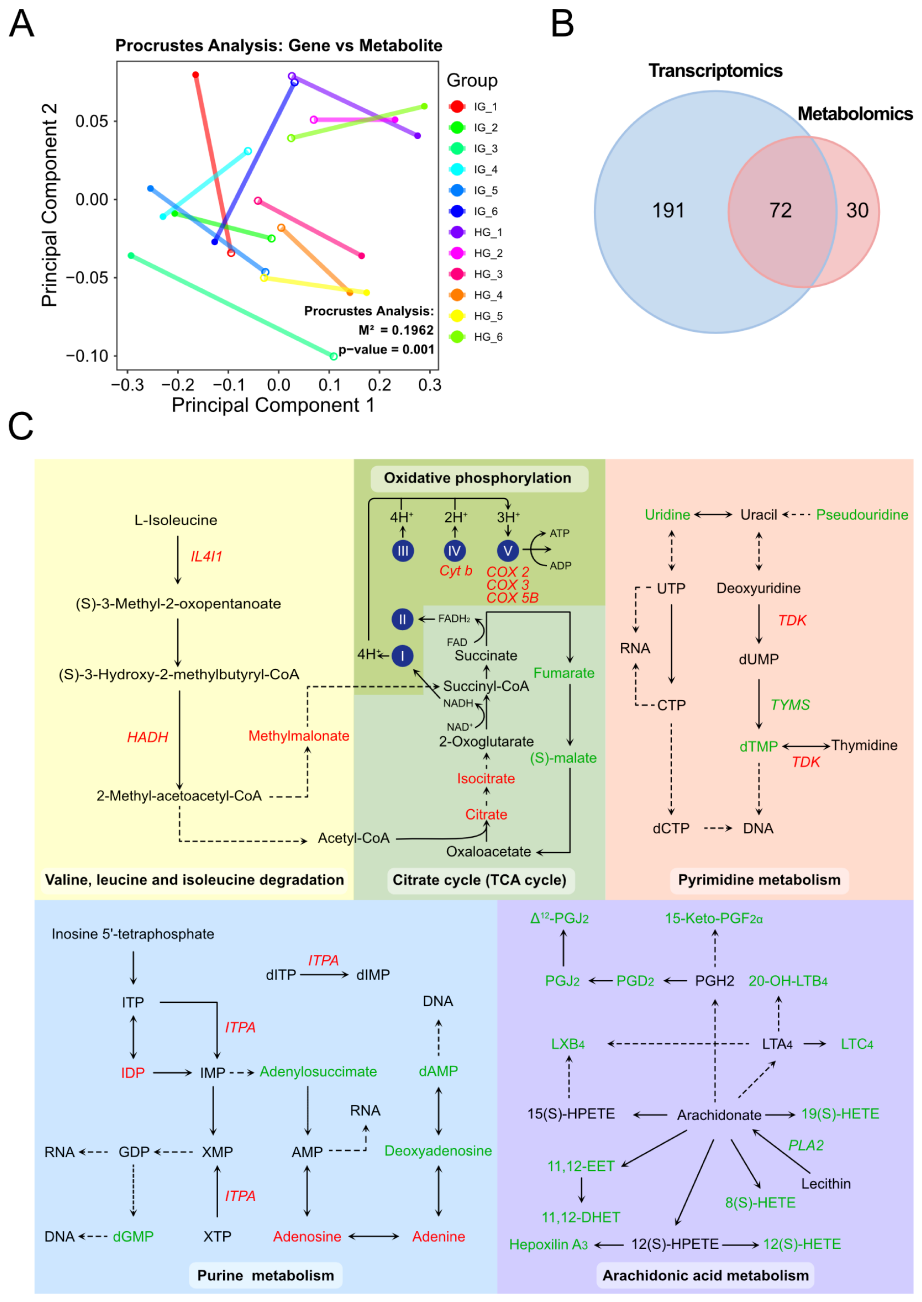
Comprehensive integrative analysis of transcriptomic and metabolomic data. (**A**) Procrustes analysis between the transcriptomic and metabolomic datasets. (**B**) Venn diagram of metabolic pathways jointly enriched by transcriptomic and metabolomic analyses. (**C**) Key exploratory regulatory networks based on candidate differentially expressed genes (DEGs) and differential metabolites (DMs). Red font indicates high expression or abundance in the HG group, while green font indicates high expression or abundance in the IG group. Genes are italicized and metabolites are in roman type. Solid arrows represent direct effects, and dashed arrows represent indirect effects. Abbreviations: *IL4I1*, L-amino acid oxidase; *HADH*, 3-hydroxyacyl-CoA dehydrogenase; *Cyt b*, cytochrome b; *COX2*, cytochrome c oxidase subunit 2; *COX3*, cytochrome c oxidase subunit 3; *COX5B*, cytochrome c oxidase subunit 5b; ATP, adenosine 5′-triphosphate; ADP, adenosine 5′-diphosphate; *TDK*, thymidine kinase; *TYMS*, thymidylate synthase; dTMP, deoxythymidylic acid; UTP, uridine 5′-triphosphate; CTP, cytidine 5′-triphosphate; dCTP, deoxycytidine 5′-triphosphate; dUMP, deoxyuridine 5′-phosphate; RNA, ribonucleic acid; DNA, deoxyribonucleic acid; IDP, inosine 5′-diphosphate; *ITPA*, inosine triphosphate pyrophosphatase; ITP, inosine 5′-triphosphate; dITP, 2′-deoxyinosine 5′-triphosphate; IMP, inosine 5′-monophosphate; dIMP, 2′-deoxyinosine 5′-phosphate; GDP, guanosine 5′-diphosphate; XMP, xanthosine 5′-phosphate; XTP, xanthosine 5′-triphosphate; dGMP, 2′-deoxyguanosine 5′-monophosphate; AMP, adenosine 5′-monophosphate; dAMP, 2′-deoxyadenosine 5′-monophosphate; PGJ_2_, prostaglandin J_2_; PGD_2_, prostaglandin D_2_; Δ^12^-PGJ_2_, Δ^12^-prostaglandin J_2_; 15-keto-PGF_2_α, 15-keto-prostaglandin F_2_α; PGH_2_, prostaglandin H_2_; LXB_4_, lipoxin B_4_; LTA_4_, leukotriene A_4_; 20-OH-LTB_4_, 20-hydroxyleukotriene B_4_; LTC_4_, leukotriene C_4_; *PLA2*, phospholipase A_2_; 19(S)-HETE, (19S)-hydroxyeicosatetraenoic acid; 8(S)-HETE, (8S)-hydroxyeicosatetraenoic acid; 11,12-EET, 11,12-epoxyeicosatrienoic acid; 11,12-DHET, 11,12-dihydroxyeicosatetraenoic acid; 12(S)-HPETE, 12(S)-hydroperoxyeicosatetraenoic acid; 12(S)-HETE, 12(S)-hydroxyeicosatetraenoic acid.

To assess the concordance between transcriptomic and metabolomic profiles of Hong Kong oysters, we performed Procrustes analysis. The results revealed a significant correlation between the gene expression and metabolite abundance datasets (M^2^ = 0.1962, *p* = 0.001), (Figure 7A), indicating high consistency between the two omics datasets. An exploratory Venn diagram analysis of pathways enriched from the 3161 candidate DEG set and the differential metabolite set identified 72 shared pathways (Figure 7B). The resulting candidate-DEG-based integrative analysis highlighted pathways potentially as-sociated with growth heterosis, including Valine, Leucine and Isoleucine Degradation, the tricarboxylic acid (TCA) cycle, Pyrimidine Metabolism, Purine Metabolism, and Arachidonic Acid Metabolism (Figure 7C).

Compared with the IG group, the HG group showed higher expression of *IL4I1* and *HADH* in the exploratory candidate analysis, along with increased abundance of methylmalonate in the Valine, Leucine and Isoleucine Degradation pathway. For the TCA cycle, the core metabolites citrate and isocitrate were significantly enriched in the HG group, indicating enhanced energy supply capacity in hybrid oysters. In the Pyrimidine metabolism pathway, the abundance of uridine and pseudouridine was decreased in the HG group, while the expression of *TDK* was higher under the exploratory candidate criterion. For purine metabolism, the levels of the core metabolites adenosine, adenine, and IDP were significantly increased in the HG group, and *ITPA* showed higher expression in the exploratory candidate analysis, whereas the levels of dGMP and dAMP were reduced. Taken together, these exploratory candidate-gene and metabolite patterns suggest that nucleotide synthesis and turnover may be associated with rapid growth in hybrid oysters. In the Arachidonic acid metabolism pathway, multiple downstream inflammatory metabolites were significantly downregulated in the HG group, including prostaglandins (Δ^12^-PGJ_2_, PGJ_2_, PGD_2_, 15-keto-PGF_2_α), leukotrienes (LTC_4_, 20-OH-LTB_4_), and other eicosanoids (12(S)-HETE, 8(S)-HETE, 19(S)-HETE, 11,12-EET, 11,12-DHET), indicating an attenuated inflammatory response in the hybrid oysters compared to the IG group.

## 3. Discussion

Heterosis describes the improved performance of hybrid progeny relative to their parental lines, and has long been utilized in aquatic breeding to boost growth rates, survival capability and stress adaptation capacity [10,34,35,36]. In this work, the HG group exhibited significantly greater shell length and shell height than the IG group six months after attachment, demonstrating remarkable growth heterosis. Similar findings have been documented in multiple bivalve species, such as *Ruditapes philippinarum*, *C. sikamea* and *Argopecten purpuratus*, where hybrid progeny consistently display superior growth-related traits [16,37,38,39]. The primary statistically robust transcriptomic result comprised 474 FDR-supported significant DEGs (233 upregulated and 241 downregulated), while a broader set of 3161 candidate DEGs was retained for exploratory analyses; metabolome analysis detected 345 differential metabolites (DMs). Clear separation between HG and IG groups was observed in both omics datasets. Such molecular variations indicate extensive transcriptional adjustment and metabolic rewiring in hybrids, which may contribute to the molecular basis of heterosis. Procrustes analysis confirmed a strong correlation between transcriptomic and metabolomic profiles (M^2^ = 0.1962, *p* = 0.001), suggesting coordinated transcriptional and metabolic changes associated with growth advantage. Multi-omics integration has become a reliable method to explore biological mechanisms behind pathogen defense, environmental acclimation and larval development [40,41,42,43,44]. The present mantle multi-omics data provide a tissue-specific perspective on growth heterosis in this oyster cross and highlight mantle-associated processes potentially linked to this phenotype.

Organismal growth is a complex quantitative trait controlled by multiple interactive genes. Faster individual development relies on active cell division, cell differentiation and efficient protein synthesis [45,46,47,48]. In this study, exploratory screening identified higher expression of several growth-related candidate genes in HG, including *MEGF10*, *MEGF11*, *GDF2*, lin-41-like and centromere protein O-like genes. MEGF10 modulates muscle stem cell proliferation and differentiation through the Notch signaling cascade [49]. In zebrafish, mutations in *MEGF10* lead to recessive congenital myopathy [50]. Consistent with our findings, 11 *MEGF10* homologs were enriched in fast-growing *Crassostrea gigas* strains [51]. As a paralog of *MEGF10*, *MEGF11* is closely linked to growth traits in livestock and reproductive performance in sows [52,53]. GDF2, also named BMP9, participates in osteogenesis, stem cell fate determination, angiogenesis and neurodevelopment [54]. LIN-41, a member of the TRIM-NHL family, acts as a downstream target of let-7 miRNA, and is a key regulator of stem cell renewal, differentiation and somatic cell reprogramming [55,56]. As a core component of the CENP-O kinetochore complex, CENP-O forms a stable assembly with CENP-P, CENP-Q and CENP-50. It maintains normal kinetochore function, ensures accurate mitotic progression, and prevents premature sister chromatid separation during spindle damage recovery [57]. Elevated expression of these growth regulators may enhance intracellular signaling and be associated with increased metabolic activity contributing to growth.

The oyster shell provides physical protection, structural support and mineral storage for soft tissues, and its growth rate directly determines the overall developmental potential of oysters [58]. The mantle tissue acts as the key functional organ governing shell biomineralization throughout the whole life cycle [59,60]. In this study, mantle samples collected from the HG and IG groups were used for transcriptome sequencing to identify biomineralization-related genes. Exploratory screening identified higher expression of multiple candidate genes associated with shell formation in HG, such as nacrein-like protein C1, spidroin-1-like, chitinase, shematrin-like protein 1, and shematrin-like protein 2. Nacrein, the first molluscan organic matrix protein found to mediate nacre formation, creates a favorable microenvironment for calcium carbonate crystal nucleation by enriching HCO_3_^−^, and acts as a core regulator of shell biomineralization [61,62]. Elevated nacrein expression is consistently observed in fast-growing bivalves [58,63]. Spidroin is a structural protein rich in alanine repeat motifs. In *Crassostrea virginica*, this protein participates in mantle-mediated mineralization and promotes shell growth [58]. Mucin is a highly glycosylated macromolecular protein rich in serine, threonine, and proline, which modulates biomineralization in mollusks [64]. Chitinase exhibits diverse biological functions; in bivalves, it not only exerts immune effects [65], but also mediates biomineralization in pearl oysters, limpets, and blunt-gaper clams [66,67,68]. Shematrin proteins are secreted by the mantle edge and deposited into the prismatic layer of the shell, forming the structural foundation for shell calcification [69]. Upregulated transcription of mineralization-related genes suggests increased potential for shell biomineralization. Rapid shell expansion creates sufficient living space for soft tissue growth, and effectively improves the overall developmental potential of hybrid oysters.

Energy metabolism supplies ATP for biological synthesis, cell proliferation and shell mineralization, and differences in energy utilization efficiency largely account for growth heterosis in shellfish [70,71,72]. In the present study, the exploratory candidate-gene and metabolite analyses suggested coordinated changes in branched-chain amino acid (BCAA) catabolism, the TCA cycle, and oxidative phosphorylation in the HG group, consistent with enhanced energy metabolism potentially supporting growth. In the valine, leucine, and isoleucine degradation pathway, the exploratory candidate analysis showed higher expression of *IL4I1* and *HADH* in HG, accompanied by increased methylmalonate production. Methylmalonate was further converted into succinyl-CoA, which replenishes carbon skeletons for the TCA cycle. This pattern was consistent with altered TCA cycle activity, accompanied by significant citrate and isocitrate accumulation in HG. Enhanced cycle activity generates abundant NADH and FADH_2_ to fuel the mitochondrial electron transport chain (ETC) [73]. Accordingly, genes encoding key ETC components, including *Cyt b*, *COX2*, *COX3* and *COX5B*, showed higher expression in HG under the exploratory candidate criterion. These subunits are key components of oxidative phosphorylation complexes involved in ATP production [74]. Notably, reduced fumarate and (S)-malate contents in HG do not indicate metabolic blockage, but reflect rapid consumption of downstream intermediates under high metabolic flux. Together, these exploratory patterns suggest enhanced energy metabolism and a potentially increased capacity for ATP production in hybrid oysters. In contrast, the IG group exhibited attenuated energy metabolism and reduced energy output. A limited energy supply restricts material allocation for growth, ultimately resulting in slow development. Enhanced energy metabolism is a conserved metabolic characteristic of fast-growing shellfish hybrids, allowing reasonable energy distribution toward growth-related physiological processes [72,75].

Nucleotide metabolism provides indispensable precursors for DNA replication, RNA transcription, and cell cycle progression, and its metabolic reprogramming has been widely recognized as a key contributor to heterosis [76,77]. Nucleotide biosynthesis occurs via de novo and salvage pathways [78,79]. In the present study, in the pyrimidine metabolism pathway, the levels of uridine, pseudouridine, and dTMP were significantly reduced in HG. In the exploratory candidate analysis, *TYMS* (the rate-limiting gene for de novo dTMP synthesis) showed lower expression, whereas the key pyrimidine salvage gene *TDK* showed higher expression. The decreased levels of uridine and pseudouridine suggest altered pyrimidine nucleoside metabolism, which may be associated with increased demands for nucleic acid synthesis during growth. The lower expression of *TYMS* in the exploratory candidate analysis, together with reduced dTMP, suggests weakened de novo synthesis, while the higher expression of *TDK* in the exploratory candidate analysis reflects a metabolic shift toward the more energy-efficient salvage pathway [80], potentially supporting deoxynucleotide availability during cell proliferation. In purine metabolism, the levels of IDP, adenosine, and adenine were significantly increased in the hybrid group, indicating enhanced turnover and interconversion of purine nucleosides to maintain a sufficient precursor pool for nucleic acid synthesis. The reduced levels of downstream deoxynucleotides, including dAMP and dGMP, suggest changes in nucleotide metabolism that may be associated with increased requirements for DNA synthesis during rapid growth. Meanwhile, the higher expression of *ITPA* in the exploratory candidate analysis, which encodes an enzyme that specifically removes noncanonical purine nucleotides [81,82], may help maintain nucleotide pool balance during DNA replication, prevent base mismatches and replication errors, and safeguard genomic stability [83]. Consistent with our findings, previous studies on *Eriocheir sinensis* and *Stichopus monotuberculatus* confirmed that fast-growing aquatic organisms exhibit lower purine and pyrimidine contents but higher nucleotide metabolic activity [24,84]. Dynamic adjustment of nucleotide metabolism is associated with an altered balance between energy consumption and cell proliferation, which may contribute to the fast-growing characteristics of hybrid oysters.

Arachidonic acid metabolism serves as a core regulatory network for immune and inflammatory responses as well as cellular homeostasis. After being released from membrane phospholipids by PLA2, arachidonic acid is metabolized via COX, LOX and CYP450 pathways to produce various bioactive lipid mediators, which participate in immune regulation and tissue repair [85,86]. Arachidonic acid metabolism is frequently activated in shellfish exposed to pathogen infection and adverse environmental stress to initiate rapid immune responses [87,88]. In the present study, the exploratory multi-omics analysis suggested lower activity of the arachidonic acid metabolic pathway in fast-growing hybrid oysters. In the exploratory candidate analysis, *PLA2*, which encodes the initial rate-limiting enzyme of this pathway, showed lower expression, accompanied by widespread reduction in downstream inflammatory metabolites. These alterations suggest changes in eicosanoid-related inflammatory metabolism that may be associated with growth performance. This physiological pattern is highly consistent with findings in fast-growing sea cucumbers, where low basal inflammatory activity has been associated with enhanced growth performance [24]. The observed alterations in arachidonic acid metabolism suggest a potential association between immune-related metabolic processes and somatic development, which may be involved in growth heterosis.

However, reduced arachidonic acid metabolism may also represent a potential trade-off between growth-oriented resource allocation and immune preparedness under environmental challenges. Previous studies in Pacific oysters (*C. gigas*) demonstrated that arachidonic acid (ARA) supplementation enhanced hemocyte phagocytosis and increased prostaglandin production, indicating that ARA-derived metabolites contribute to oyster immune activation [89,90]. Similarly, activation of ARA metabolism was observed in *Apostichopus japonicus* following *Vibrio splendens* infection, suggesting its involvement in pathogen-induced immune responses [91]. Moreover, thermal stress has been shown to dynamically regulate ARA-derived lipid mediators in marine bivalves, highlighting the potential role of this pathway in stress adaptation [92]. Therefore, the reduced basal arachidonic acid metabolism observed in hybrid oysters may reflect an alternative metabolic strategy that supports growth under normal culture conditions; however, whether this alteration influences immune responsiveness and stress adaptation during pathogen exposure or high-temperature stress requires further investigation.

Comparisons with previous studies suggest that several mechanisms identified here are shared among oysters and other bivalves. Accelerated aerobic metabolism and activation of the TCA cycle were associated with growth heterosis in hybrid *C. ariakensis* [32], while growth heterosis in *C. gigas* involved energy metabolism, cell proliferation, biomineralization, and innate immunity [65]. In pearl oyster *Pinctada fucata martensii*, purine metabolism and immune- and metabolism-related pathways were associated with heterosis [68,93], and fast-growing individuals exhibited greater biomineralization and anabolic activity while allocating less energy to stress responses [22]. Similar energy-mediated growth–defense trade-offs involving purine, pyrimidine, and arachidonic acid metabolism have also been reported in *C. gigas* and *C. angulata* [94]. These overlaps indicate that enhanced energy production, nucleotide turnover, biomineralization, and resource reallocation between growth and immunity may represent conserved features of bivalve growth heterosis. However, their coordinated occurrence with branched-chain amino acid degradation and broad suppression of inflammatory eicosanoids may represent a potentially species- or cross-specific feature of hybrid *C. hongkongensis*.

Overall, the results support an association between growth heterosis and coordinated, mantle-associated transcriptional and metabolic changes involving energy metabolism, biosynthetic processes, shell biomineralization, and immune-related pathways. Although the 474 FDR-supported significant DEGs were treated as the primary statistically robust transcriptomic findings, the broader set of 3161 candidate DEGs identified using *p* < 0.05 and |log_2_FC| ≥ 1 was used for pathway enrichment and multi-omics integration analyses to provide broader biological context. Accordingly, these pathway-level analyses are interpreted as exploratory complements to the FDR-supported findings and highlight biological processes potentially associated with growth heterosis. In addition, the present study mainly reflects changes in gene expression and metabolite abundance. Therefore, the identified metabolic pathways should be interpreted as potential mechanisms rather than direct evidence of altered metabolic flux or causal regulation. Further studies involving enzymatic activity assays, physiological measurements, and metabolic flux analyses are required to validate these proposed mechanisms and clarify the regulatory basis of growth heterosis.

In addition, because each omics replicate was generated by pooling mantle tissue from three oysters, the present experimental design characterizes variation among independent composite samples rather than variation among individual oysters. Pooling may average individual-specific molecular signals and reduce the apparent within-group variability, thereby limiting the direct assessment of inter-individual molecular variation. Accordingly, the transcriptomic and metabolomic findings should be interpreted primarily as population-level molecular signatures in mantle tissue that are associated with growth heterosis, rather than as individual-level molecular responses. Furthermore, because both omics analyses were restricted to mantle tissue, the identified molecular changes mainly represent mantle-associated responses and should not be generalized to organism-wide growth regulation or metabolic adaptation. Whether comparable regulatory patterns occur in gill, muscle, or other tissues remains to be determined. Future studies using unpooled samples from individual oysters across multiple tissues will be required to quantify inter-individual and tissue-specific variation and determine whether the identified molecular signatures are consistently observed across individuals and tissues.

## 4. Materials and Methods

### 4.1. Parental Populations, Mating Design, and Sample Preparation

Experimental oysters were generated from the Qinzhou (Q) population in Guangxi Province and the Shanwei (S) population in Guangdong Province and reared at the Yangjiang Base of the Sanya Tropical Fisheries Research Institute. A 2 × 2 complete diallel crossing design was conducted between the two populations. For each population, 300 sexually mature females and 300 sexually mature males were used. Eggs and sperm were obtained by gonadal dissection and pooled separately within each population. Each pooled gamete sample was then divided into two portions to generate two intra-population groups, Q♀ × Q♂ (QQ) and S♀ × S♂ (SS), and two reciprocal inter-population hybrid groups, Q♀ × S♂ (QS) and S♀ × Q♂ (SQ). Each progeny group therefore represented a mass-spawned cohort derived from pooled gametes of multiple parents rather than a single full-sib family. During settlement, cement plates were used as settlement substrates for the larvae. Within 10 days after settlement, the cement plates bearing spat from the four progeny groups were transferred to the same grow-out pond, where all groups were reared under the same environmental and management conditions. At six months post-settlement, 36 oysters were randomly selected from each of the four mating groups, and shell length and shell height were measured in 30 individuals per group. Following phenotypic evaluation of all four mating groups (Figure S1), SQ and QQ were selected as the representative inter-population hybrid group (HG) and intra-population group (IG), respectively, for subsequent multi-omics analyses aimed at characterizing the molecular and metabolic changes associated with growth heterosis. All 36 oysters from each selected group were then anesthetized and euthanized for tissue collection. For each selected group, equal amounts of mantle tissue from every three oysters were pooled to generate one composite sample, yielding 12 composite samples per group. Each composite sample represented three independent oysters and was treated as one biological replicate. The 12 composite samples were randomly divided into two non-overlapping sets: six biological replicates were used for transcriptome sequencing, and the remaining six were used for metabolomic analysis. Thus, the transcriptomic and metabolomic analyses were conducted using independent composite samples derived from the same experimental groups. Accordingly, the experimental unit for both transcriptomic and metabolomic analyses was the three-oyster composite sample (n = 6 independent composite samples per group for each omics platform), rather than the individual oyster. Therefore, the estimated statistical variation reflects differences among independent composite samples and does not directly represent molecular variation among individual oysters. All samples were immediately snap-frozen in liquid nitrogen and stored at −80 °C until further analysis.

### 4.2. RNA Extraction, Library Construction and High-Throughput Sequencing

Total RNA was extracted from frozen mantle tissue samples using TRIzol reagent (Invitrogen, Carlsbad, CA, USA), followed by DNase I treatment to remove residual genomic DNA. We performed three parallel quality control assays for the eluted RNA: concentration and purity were measured on a NanoDrop 2000 spectrophotometer (Thermo Fisher Scientific, Waltham, MA, USA), integrity was validated by 1% non-denaturing agarose gel electrophoresis, and the RNA Quality Number (RQN) was determined using an Agilent 5300 Bioanalyzer (Agilent Technologies, Santa Clara, CA, USA). Only RNA samples that passed all quality thresholds were advanced to library construction, with the inclusion criteria as follows: total RNA yield ≥ 1 μg, minimum concentration of 30 ng/μL, RQN value > 6.5, and OD_260_/_280_ ratio between 1.8 and 2.2. For library preparation, poly(A)-tailed mRNA molecules were first captured from qualified total RNA via oligo(dT)-conjugated magnetic beads, and then chemically fragmented into ~300 bp short fragments using fragmentation buffer. Reverse transcription was performed with random hexamer primers to synthesize first-strand DNA (cDNA), followed by second-strand synthesis to generate stable double-stranded cDNA (ds cDNA). The ds cDNA fragments were then processed through sequential enzymatic reactions: end repair to generate blunt ends using End Repair Mix, 3′ adenylation to add a single adenine overhang, and ligation of dual-index sequencing adapters to the fragment ends. Adapter-ligated products were purified with magnetic beads and size-selected to recover target fragments, which were then amplified by PCR. The final purified PCR products constituted the sequencing-ready cDNA libraries, which were subjected to high-throughput paired-end sequencing on the Illumina NovaSeq X Plus platform (Illumina, Inc., San Diego, CA, USA).

### 4.3. Transcriptome Analysis

Transcriptome sequencing data were analyzed using a de novo transcriptome assembly strategy. Raw sequencing reads were preprocessed using fastp software (v0.23.2) to generate high-quality clean reads. During this process, adapter sequences, low-quality reads, reads with ambiguous base (N) content exceeding 10%, and sequences shorter than 20 bp after quality trimming were filtered out. De novo transcriptome assembly of all clean reads was performed using Trinity (v2.15.1) with default parameters. The contigs obtained from the assembly were filtered and optimized using TransRate (v1.0.3), and redundant nucleic acid sequences were subsequently removed using CD-HIT (v4.8.1). The completeness of the final assembled transcriptome was evaluated using the Benchmarking Universal Single-Copy Orthologs (BUSCO v5.6.0) pipeline. For functional annotation, the assembled transcripts were aligned against six public databases (NR, Swiss-Prot, Pfam, COG, GO, and KEGG) using Diamond (v2.1.8) and HMMER (v3.3.2). The non-redundant transcriptome database generated after CD-HIT filtering was used as the reference database, and gene and transcript expression levels were estimated using RSEM (v1.3.3) with default parameters. The resulting read count matrix was used for differential gene expression analysis using DESeq2 (v1.40.2). Candidate DEGs were defined for exploratory screening using a *p* value < 0.05 and |log_2_FC| ≥ 1. Benjamini–Hochberg correction was then applied across all tested transcripts. Genes with an FDR-adjusted *p* value < 0.05 and |log_2_FC| ≥ 1 were considered statistically significant and were prioritized as the primary robust transcriptomic findings; the complete candidate DEG list was retained in Table S2. Exploratory Gene Ontology (GO) and Kyoto Encyclopedia of Genes and Genomes (KEGG) pathway enrichment analyses were performed on the 3161 candidate DEG set using Goatools (v1.2.3) and the Python SciPy package (v1.9.3), respectively.

### 4.4. RT-qPCR Validation of Differential Gene Expression

To verify the accuracy and reproducibility of the transcriptome sequencing data, 11 candidate differentially expressed genes (DEGs) were randomly selected for quantitative real-time PCR (RT-qPCR) validation. Gene-specific primers were designed using Primer Premier 5.0. Amplification efficiencies were determined from standard curves generated using serial dilutions of pooled cDNA, and the primer sequences and amplification efficiencies are provided in Table 1. Total RNA was isolated from the same batch of mantle tissue used for RNA-seq to ensure assay consistency. cDNA was synthesized via reverse transcription with random hexamer primers using the TaKaRa PrimeScript™ RT Reagent Kit with gDNA Eraser (Takara, Dalian, China) per the manufacturer’s protocol, with residual genomic DNA removed to eliminate interference. RT-qPCR was performed on a LightCycler^®^ 480 System (Roche Diagnostics, Indianapolis, IN, USA) using TB Green^®^ Premix Ex Taq™ II (Takara, Dalian, China), with parameters optimized according to the manufacturer’s instructions. For each group, six independent biological replicates were analyzed, with three technical replicates performed for each biological replicate. Thermal cycling conditions: 95 °C for 30 s, followed by 40 cycles of 95 °C for 5 s and 60 °C for 30 s. Melting curve analysis was conducted post-amplification to confirm amplicon specificity. *β-actin* and *GAPDH* were selected as reference genes based on previous studies of *C. hongkongensis* [95,96]. Both genes showed stable cycle threshold (Ct) values across all samples, with standard deviations of 0.53 and 0.63 and coefficients of variation of 3.96% and 3.65%, respectively. Their Ct values did not differ significantly between the IG and HG groups (*β-actin*, *p* = 0.996; *GAPDH*, *p* = 0.997). Therefore, the geometric mean of *β-actin* and *GAPDH* expression was used for normalization. The three technical replicates were averaged for each biological replicate, and relative expression was calculated using the 2^−ΔΔCt^ method [97]. Differences between the IG and HG groups were evaluated using a two-tailed Welch’s *t*-test based on the ΔCt values from six biological replicates per group. Data are presented as the mean ± standard deviation (SD), with *p* < 0.05 considered statistically significant. Pearson’s correlation analysis was performed between the RNA-seq and RT-qPCR log_2_ fold change values of the 11 validated genes.

### 4.5. Metabolite Extraction and Data Analysis

Non-targeted LC-MS/MS metabolomic profiling was performed on mantle tissues of *C. hongkongensis*, with six pooled biological replicates established for each experimental group. Each pooled biological replicate was generated by mixing equal amounts of mantle tissues from three individual oysters. Briefly, 50 mg of frozen mantle tissue was accurately weighed and mixed with 400 μL of pre-cooled methanol-water extraction solution (4:1, *v*/*v*) and a 6 mm grinding bead. The mixture was homogenized in a cryogenic grinder at −10 °C and 50 Hz for 6 min, followed by ultrasonic-assisted extraction at 40 kHz and 5 °C for 30 min. The homogenate was incubated at −20 °C for 30 min to facilitate protein precipitation and centrifuged at 13,000× *g* for 15 min at 4 °C. The supernatant was collected and transferred into sample vials with inner liners for LC-MS/MS analysis.

Metabolomic analysis was performed using a UHPLC-Q Exactive HF-X mass spectrometry platform (Thermo Fisher Scientific, Waltham, MA, USA). Chromatographic separation was achieved using an ACQUITY UPLC HSS T3 column (100 mm × 2.1 mm i.d., 1.8 μm; Waters Corporation, Milford, MA, USA), maintained at 40 °C. The mobile phase A consisted of 95% water and 5% acetonitrile containing 0.1% formic acid, while mobile phase B consisted of 47.5% acetonitrile, 47.5% isopropanol, and 5% water containing 0.1% formic acid. The injection volume was 3 μL. Metabolites were ionized using an electrospray ionization (ESI) source, and data were acquired in both positive and negative ion modes. The optimized MS conditions were as follows: source temperature, 425 °C; sheath gas flow rate, 50 arb; auxiliary gas flow rate, 13 arb; ion spray voltage floating (ISVF), 3500 V in positive mode and −3500 V in negative mode; normalized collision energy, 20, 40, and 60 V. Data acquisition was performed using the data-dependent acquisition (DDA) mode over a mass range of 70–1050 m/z.

Quality control (QC) samples were prepared by pooling equal volumes of extraction supernatants from all pooled biological samples. QC samples were processed and analyzed using the same procedure as experimental samples and were inserted every 5–15 analytical samples throughout the sequence to monitor the stability and reproducibility of the LC-MS/MS system.

Raw LC-MS/MS data were processed using Progenesis QI v3.0 software (Waters Corporation, Milford, MA, USA). Data preprocessing included baseline filtering, peak detection, peak integration, retention time correction, and peak alignment. A data matrix containing retention time, mass-to-charge ratio (m/z), and peak intensity information was generated. Metabolite annotation was performed by matching MS and MS/MS spectral information against metabolite databases, including HMDB and Metlin. The mass error tolerance for MS matching was set to <10 ppm, and metabolite identification confidence was evaluated based on accurate mass matching and MS/MS spectral similarity.

The processed data matrix was further analyzed using the Majorbio Cloud platform (Majorbio Bio-Pharm Technology Co., Ltd., Shanghai, China). Missing values were filtered according to the 80% rule, retaining variables with non-zero values in at least 80% of samples within each experimental group. Remaining missing values were imputed using the minimum detected value of the original dataset. Peak intensities were normalized using sum normalization to minimize variations caused by sample preparation and instrumental instability. Variables with relative standard deviation (RSD) values > 30% among QC samples were removed. The normalized data matrix was subsequently subjected to log10 transformation before downstream statistical analyses.

Principal component analysis (PCA) and orthogonal partial least squares discriminant analysis (OPLS-DA) were performed using the R package “ropls” (version 1.42.0). The PCA, OPLS-DA, and permutation analyses were performed using this combined dataset. Seven-fold cross-validation was used to estimate model performance. The cumulative R^2^Y value [R^2^Y(cum)] was used to describe model goodness of fit, whereas the cumulative Q^2^ value [Q^2^(cum)] was used to evaluate predictive performance. A permutation test with 200 random permutations of the group labels was conducted to evaluate model significance and potential overfitting. The original R^2^Y and Q^2^ values were compared with their corresponding permutation distributions. Model performance was considered supported when the original R^2^Y and Q^2^ values exceeded their corresponding permuted values and the permutation-derived *p* values were <0.05. Predictive performance was further evaluated based on the Q^2^ regression intercept, with a negative intercept indicating that the predictive performance of the original model was superior to that of the permuted models. Because R^2^Y primarily reflects goodness of fit, the R^2^Y intercept was reported as an additional diagnostic and was not used alone to evaluate predictive validity. Differential metabolites (DMs) were identified based on the variable importance in projection (VIP) values obtained from the OPLS-DA model and Student’s *t*-test *p*-values. Metabolites with VIP values > 1 and *p*-values < 0.05 were considered differential metabolites. Functional annotation of differential metabolites was performed using the KEGG database, and pathway enrichment analysis was conducted using the “scipy.stats” package in Python.

### 4.6. Statistical Methods for Integrative Analysis of Transcriptomic and Metabolomic Data

Procrustes analysis was performed using the vegan package (version 2.7.5) in R software (version 4.5.2) to evaluate the concordance between transcriptomic (3161 candidate DEGs) and metabolomic (DMs) datasets. The M^2^ value and *p*-value were calculated to determine the significance of the correlation between the two omics datasets. Exploratory Venn diagram analysis of pathways enriched from the candidate DEG and differential metabolite sets was performed using the VennDiagram package (version 1.8.2) in R software to identify shared enriched pathways.

## 5. Conclusions

In this study, integrated transcriptomic and metabolomic analyses of mantle tissue were used to characterize molecular signatures associated with growth heterosis in hybrid *C. hongkongensis*. The inter-population hybrid group exhibited significantly greater shell dimensions than the intra-population group and showed distinct transcriptional and metabolic profiles in mantle tissue. The data revealed associations involving genes related to cell proliferation and shell biomineralization, together with pathways related to energy metabolism, nucleotide metabolism, and arachidonic acid metabolism. Because the analyses were based on pooled samples from a single tissue, these findings should be interpreted as population-level, mantle-associated molecular signatures rather than organism-wide mechanisms or individual-level responses. Whether comparable regulatory patterns occur in gill, muscle, or other tissues remains to be determined. Future studies using individual samples, multiple tissues, and functional validation will be required to establish the causality and generality of the identified molecular signatures. Nevertheless, this study provides candidate mantle-associated genes and pathways for further investigation and for the genetic improvement of fast-growing oyster strains.

## Figures and Tables

**Table 1:** Primers for RT-qPCR gene expression analysis.

Primer Name	Primer Sequence (5′-3′)	Efficiency (%)
chitinase-F	AACAGTGTATGACGAGGAGCA	96.3
chitinase-R	AAATGTAAGGAGTGATGTGGAAT	
angiopoietin-4-like-F	ATGAATGAACAAGTGCTGAGTG	97.7
angiopoietin-4-like-R	CTGTCTTAGGAGCCGTTGC	
cerebellin-1-like-F	GCTCACTGGGCGAGATGG	98.2
cerebellin-1-like-R	GGAGGACTTGTAAGCAGAAAAC	
toll-like receptor 4-F	CTTGATAGGAAGTCTTTGGTCTG	99.1
toll-like receptor 4-R	ATTTCACTGGTGGAATGGAGA	
cystatin-A-like-F	CTTGACCTGGTAGTAAGTGCCC	97.5
cystatin-A-like-R	TTTGAAGAGTGACATTGTGAGTAGA	
sacsin-like-F	AAACTCAGGTGCTATGGATGG	99.6
sacsin-like-R	TTGCTGTAAAAGGTTGTGGAT	
fibrocystin-L-like-F	CGTGACCTCTGTCCCTGCTGA	97.2
fibrocystin-L-like-R	ATCTGGGTGCTGCTGTTGCTG	
hemicentin-2-like-F	ATCCTGCCCCGAAGCGTAT	96.4
hemicentin-2-like-R	TCTGAGATTCTGTTTCCCACC	
deleted in malignant brain tumors 1 protein-like-F	CCCTTGTTACCCAAATCCTT	98.4
deleted in malignant brain tumors 1 protein-like-R	GCTACATTCCAGTTGTTCTCG	
eppin-like-F	TTGTAATCCGCAGCGTCAT	96.7
eppin-like-R	GGGGTTTTGTGGTGCCTTT	
tetraspanin-1-like-F	ATGACAGCCACTTGGAGCC	96.2
tetraspanin-1-like-R	TGGTGTACCACTTCGGGATC	
GAPDH-F	GGATTGGCGTGGTGGTAGAG	99.1
GAPDH-R	GTATGATGCCCCTTTGTTGAGTC	
β-actin-F	CTGTGCTACGTTGCCCTGGACTT	99.6
β-actin-R	TGGGCACCTGAATCGCTCGTT	

## Data Availability

The original RNA-seq data presented in the study are openly available in the NCBI Sequence Read Archive (SRA) and are publicly available under BioProject accession number PRJNA1478274 at https://www.ncbi.nlm.nih.gov/bioproject/PRJNA1478274, accessed on 20 July 2026. The raw metabolomics data are available from the corresponding author upon reasonable request.

## Supplementary Materials

The following supporting information can be downloaded at: https://www.mdpi.com/article/10.3390/ijms27156631/s1.
