# Supplementary material for: Transcriptomic and Metabolomic Insights into Growth Heterosis of Crassostrea hongkongensis Hybrids
Source: Int J Mol Sci. 2026 Jul 25;27(15):6631. doi: 10.3390/ijms27156631 (PMC13465682; doi:10.3390/ijms27156631)
Supplement: Supplementary file 1 [file ijms-27-06631-s001.zip › Figure S1.pdf]

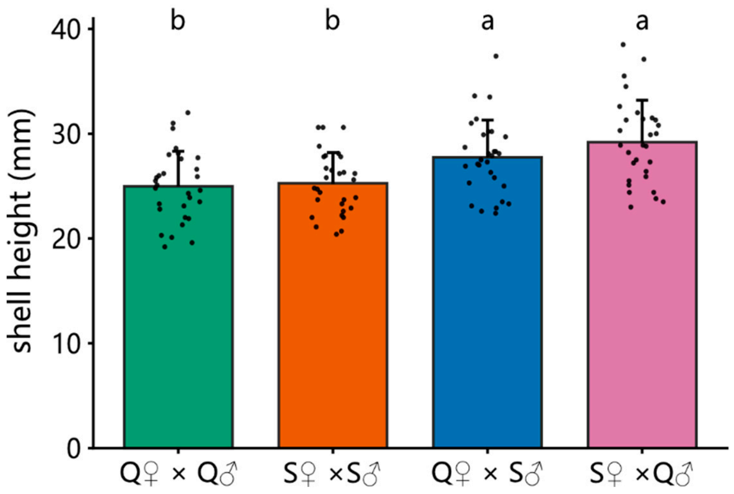

Figure S1. Shell height of the four mating groups generated by the 2 × 2 complete diallel crossing design in *C. hongkongensis* at six months post-settlement. Data are presented as the mean ± SD (n = 30). Different lowercase letters indicate significant differences among groups based on one-way ANOVA followed by Tukey's multiple-comparison test ($p < 0.05$).
