# Supplementary material for: Transcriptomic and Metabolomic Insights into Growth Heterosis of Crassostrea hongkongensis Hybrids
Source: Int J Mol Sci. 2026 Jul 25;27(15):6631. doi: 10.3390/ijms27156631 (PMC13465682; doi:10.3390/ijms27156631)
Supplement: Supplementary file 1 [file ijms-27-06631-s001.zip › Table S1.pdf]

Table S1. Summary of the transcriptome assembly.

Sample	Raw reads	Raw bases	Clean reads	Clean bases	Q20(%)	Q30(%)	GC content (%)	Mapped ratio
IG_1	36553116	5519520516	36370338	5467372479	99.42	96.97	43.56	86.42%
IG_2	38374478	5794546178	38171490	5741786950	99.42	96.98	43.22	86.25%
IG_3	39480424	5961544024	39273746	5894993657	99.42	96.97	43.42	87.40%
IG_4	42615252	6434903052	42396398	6374183595	99.43	97.03	43.38	87.00%
IG_5	36281952	5478574752	36067624	5419686586	99.43	97.04	43.38	86.72%
IG_6	39444086	5956056986	39209102	5890959992	99.41	96.94	43.00	85.51%
HG_1	38055632	5746400432	37814634	5678593142	99.42	97.01	43.17	86.31%
HG_2	37629102	5681994402	37409290	5617141855	99.37	96.79	44.28	86.78%
HG_3	40740396	6151799796	40502208	6083975385	99.42	97.00	43.29	86.36%
HG_4	38754526	5851933426	38524542	5790010207	99.42	96.97	43.52	86.27%
HG_5	38816848	5861344048	38577712	5793695017	99.39	96.93	43.43	86.40%
HG_6	36537136	5517107536	36353482	5456647179	99.4	96.88	44.18	86.40%
